# Supplementary material for: Localization of Epigenetic Markers in Leishmania Chromatin
Source: Pathogens. 2022 Aug 18;11(8):930. doi: 10.3390/pathogens11080930 (PMC9413968; doi:10.3390/pathogens11080930)
Supplement: Supplementary file 1 [file pathogens-11-00930-s001.zip › pathogens-1871783-supplementary.pdf]

Table S1: LtaP_2016 chromosomes and genes

	Sequence					Genes			
**Chromosome**	**Length**	**Gaps**	**CDSs**	**pseudogenes**	**rRNAs**	**slRNAs**	**snoRNAs**	**snRNAs**	**tRNAs**
01	293,541	0	92						
02	358,489	0	100			46			
03	374,440	0	95						2
04	436,848	0	143						
05	487,637	0	130		1		26		1
06	518,584	0	143						1
07	563,013	0	159						1
08	441,906	0	125						
09	567,671	0	198		3				9
10	654,149	2	307						2
11	547,029	0	153		3				7
12	636,574	2	166						
13	648,292	0	178						
14	592,770	0	160						
15	627,951	1	184						2
16	669,457	0	210						1
17	688,786	0	198						3
18	730,895	0	167						
19	661,934	0	183						
20	685,981	0	178						
21	773,031	1	247		2				4
22	704,081	0	189						
23	737,501	0	204		1				10
24	869,383	0	280					1	6
25	884,399	0	282						
26	1,068,160	0	296						
27	1,180,796	4	296		16				
28	1,165,224	0	340	1					1
29	1,188,875	0	304						2
30	1,338,345	0	419						1
31	1,694,416	2	407					1	6
32	1,531,139	0	432	1					1
33	1,513,194	2	395		1				6
34	1,679,285	0	458						11
35	1,982,148	0	572	2					
36	2,696,151	0	778						12
**Total**	**32,192,075**	**14**	**9168**	**4**	**27**	**46**	**26**	**2**	**89**

Table S2. *L. tarentolae* centromeres.

Chr	location	J-peak	type	AT-rich	RNA genes
1	270K	1.2	iTTS/TSS	Y	
2	230K	2.2	dTSS	Y	slRNA
3	240K	3.2	iTTS/TSS	Y	tRNA?
4	115K	4.2	cTTS	(Y)	
**5**	367K	5.3	cTTS	Y	tRNAs
6	124K	6.2	dTSS	Y	
7	210K	7.3	dTSS	(Y)	
8	370K	8.3	dTSS	Y	
9	270K	9.2	cTTS	Y	tRNAs/5S rRNA
10	527K	10.3	iTTS/TSS	Y	
11	169K	11.2	iTTS/TSS	Y	tRNAs/5S rRNA
12	287K	12.5	dTSS	Y	
13	142K	13.2	dTSS	Y	
14	158K	14.2	cTTS	Y	
15	335K	15.5	cTTS	Y	tRNAs
16	337K	16.2	dTSS	Y	
17	371K	17.5	iTTS/TSS	Y	tRNAs
18	461K	18.2	iTTS/TSS	(Y)	
19	603K	19.3	iTTS/TSS	Y	
20	484K	[20.3]	iTTS/TSS	Y	
21	222K	21.3	dTSS	Y	
22	605K	22.4	dTSS	(Y)	
23	529K	23.5	dTSS	Y	
24	470K	[24.3]	iTTS/TSS	Y	
25	576K	25.3	iTTS/TSS	Y	
26	592K	26.4	iTTS/TSS	Y	
27	992K	27.5	dTSS	Y	rRNAs
28	825K	28.6	dTSS	Y	
29	335K	29.2	iTTS/TSS	Y	
30	235K	30.2	dTSS	Y	
31	806K	31.6	iTTS/TSS	(Y)	
32	1175K	32.4	dTSS	Y	
33	676K	33.4	iTTS/TSS	Y	rRNA
34	310K	34.2	cTTS	Y	
35	368K	35.3	iTTS/TSS	Y	
36	1071K	36.6	iTTS/TSS	(Y)	

Table S3. Illumina sequencing libraries used in this study.

Library	Type	Description	Read pairs	Reads aligned
JM245	ATAC-seq	Chromatin (1×10^7 cells)	15,400,176	18,482,265
JM246	ATAC-seq	Chromatin (2×10^7 cells)	13,927,507	16,437,621
JM247	ATAC-seq	Genomic DNA	23,146,209	37,159,619
JM265	CUT&Tag	Anti-H2A	29,130,416	39,394,805
JM266	CUT&Tag	Anti-H2A.Z	24,059,103	32,867,982
JM267	CUT&Tag	Anti-H2B	11,462,422	12,207,997
JM268	CUT&Tag	Anti-H2B.V	16,883,972	20,471,031
JM270	CUT&Tag	Anti-H3V	13,422,639	16,013,889
JM271	CUT&Tag	Anti-H3K4me3	15,184,278	21,293,837
JM272	CUT&Tag	Anti-H3K16ac	16,056,466	20,339,157
JM273	CUT&Tag	Anti-H3K36me3	12,432,560	12,318,025
JM274	CUT&Tag	Anti-H3K50ac	3,270,369	3,078,271
JM275	CUT&Tag	Anti-H3K20me2	10,657,268	10,955,351
JM276	CUT&Tag	Anti-H3K76me3 (*T. brucei*)	3,867,726	4,666,603
JM277	CUT&Tag	Anti-H4	183,363	253,271
JM278	CUT&Tag	Anti-H3 (*T. brucei*)	92,468,418	153,273,517
JM279	CUT&Tag	Anti-HRP	7,881,445	7,987,290
JM442	J-IPseq	Anti-J	17,485,977	14,993,470
JM443	J-IPseq	No antibody	24,973,064	31,796,395
